# Critical Reappraisal of Takotsubo Syndrome (TS) and Myocarditis Association: “The Myocarditis-Like Features” Seen in TS Are Secondary Changes and not True Myocarditis

**DOI:** 10.31083/RCM46256

**Published:** 2026-02-26

**Authors:** Shams Y-Hassan

**Affiliations:** ^1^Department of Cardiology, Karolinska Institute and Karolinska University Hospital, 141 86 Stockholm, Sweden

**Keywords:** takotsubo syndrome, autonomic neurocardiogenic syndrome, acute myocarditis, coagulative myocytolysis, contraction band necrosis

## Abstract

Takotsubo syndrome (TS) is an acute cardiac disease entity characterized by a reversible regional, usually circumferential, left ventricular wall motion abnormality in patients presenting with a clinical picture resembling that of acute coronary syndrome with non-obstructive coronary arteries. Overwhelming evidence supports the involvement of sympathetic nervous system hyperactivation in the pathogenesis of TS. Therefore, the diagnostic pathogenic term of autonomic neurocardiogenic (ANCA) syndrome has also been introduced. An association between TS or ANCA syndrome and “myocarditis” has been reported. The definitive histopathological diagnosis of acute “myocarditis” is based on myocardial infiltration with mainly mononuclear cells and signs of non-ischemic myocyte necrosis with or without fibrosis. The radiological diagnosis of myocarditis is based on the cardiac magnetic resonance (CMR) imaging findings of hyperemia, myocardial oedema, and non-ischemic myocardial necrosis/fibrosis. These endomyocardial biopsy and CMR imaging findings may also be seen in TS or ANCA syndrome and have been interpreted as true “myocarditis”. However, histopathological changes in TS or ANCA syndrome begin with hypercontraction of sarcomeres, and myocardial cells may die in a tonic state if hypercontraction is severe and persistent. This myocardial cell necrosis elicits mononuclear cell infiltration, followed by fibrosis and scarring. Mononuclear cell infiltration occurs as a response or secondary process following the development of myocardial cell necrosis. Regrettably, these histopathological “secondary myocarditis-like changes” and the consequent CMR imaging findings have been, and at times remain, misdiagnosed as true “myocarditis” for many decades. These misinterpretations have been critically reviewed, analyzed, and illustrated with revealing images and with a novel conclusion.

## 1. Introduction

The terms tsubo- or takotsubo-shaped were introduced in the early 1990s to describe the left ventricular mid-apical ballooning pattern during systole in patients presented with a clinical picture of acute myocardial infarction (MI) and non-obstructive coronary arteries [1, 2]. The disease is currently known as takotsubo syndrome (TS) [3, 4]. Apart from the takotsubo-shaped left ventricle during systole, TS has other characteristic clinical features as a history of an emotional (grief in broken heart or fright in Voodoo death) or a physical trigger factor; a clinical presentation in the form of pulmonary oedema and explicitly neurogenic pulmonary oedema in acute cerebral diseases and cranial trauma; repolarization electrocardiographic (ECG) changes; “myocardial-cell necrosis” biomarker elevations; the characteristic histopathological changes of contraction band necrosis (also known as coagulative myocytolysis or myofibrillar degeneration) [5, 6]. It is worth noting that these distinctive features had been described for decades, and some of them hundreds of years before the term takotsubo was introduced [7, 8, 9, 10, 11]. Overwhelming evidence indicates that the sympathetic nervous system, including local cardiac sympathetic hyperactivation with excessive local norepinephrine release and spillover at cardiac sympathetic nerve terminals, causes the disease and, therefore, the introduction of the pathogenic diagnostic term, autonomic neurocardiogenic (ANCA) syndrome [5]. It is important to be aware that ANCA syndrome may present with a combination of phenotypes, including the takotsubo phenotype, and may do so without the takotsubo phenotype [12, 13]. A typical example is what happens in patients with acute subarachnoid hemorrhage (SAH), which is an important trigger factor for TS [12], where 70% may have left ventricular diastolic dysfunction, 67% may have ECG changes including repolarization ECG changes, 30% may have troponin elevation [13], and only 20% may have left ventricular wall motion abnormality (LVWMA) [12, 13]. For this reason, both the descriptive diagnostic term of TS and the pathophysiologic diagnostic term of ANCA syndrome are used in this presentation. In a 2022 review article, De Gregorio *et al*. [14] appropriately described TS as “a well-known disease, but not everything is clear yet”. One important such point, which remains unclear in TS and needs careful discussion, is the association between TS and myocarditis. Infectious myocarditis is among the physical diseases that may trigger TS; meanwhile, various virus-caused myocarditis types, including COVID-19, have been reported to trigger TS [15, 16, 17].

However, apart from infectious myocarditis-triggered TS, an association between TS or ANCA syndrome and myocarditis has also been reported [18, 19, 20, 21, 22]. Myocarditis is defined clinically and pathologically as an inflammation of the myocardium. The definitive histopathological diagnosis of acute myocarditis is based on myocardial infiltration by predominantly mononuclear cells and signs of non-ischemic myocyte necrosis, with or without fibrosis [23, 24]. The radiological diagnosis is based on the cardiac magnetic resonance (CMR) imaging findings: T2-based criterion with (global or regional increase of myocardial T2 relaxation time or an increased signal intensity in T2-weighted CMR-images) and with at least one T1-based criterion (increase myocardial T1, increased extracellular volume (ECV), or late gadolinium enhancement (LGE)) [23, 24]. Notably, the same cardiac histopathological changes at certain time points in the disease process and CMR imaging findings have been reported in patients with TS or ANCA syndrome with or without TS phenotype [21, 25, 26, 27]. Since the above-mentioned changes meet all diagnostic criteria for myocarditis, the condition has been misinterpreted as “myocarditis” in many reports [18, 19, 20, 21, 22, 23], leading to the erroneous exclusion of the TS diagnosis for decades. Therefore, the fundamental question remains whether this is a true “myocarditis” or merely a misinterpretation.

In this critical review, two key points are presented: First, substantial evidence is provided that the histopathological and CMR imaging findings of “myocarditis-like manifestation” seen in TS or ANCA syndrome have been deemed as true “myocarditis” before and during the era of TS. The “myocarditis” diagnosis has, in turn, resulted in the exclusion of the diagnosis of TS. Second, sufficient evidence is provided that the “myocarditis-like features” observed in TS or ANCA syndrome are secondary changes that occur only at certain stages of the disease process; the true “myocarditis” diagnosis is a misinterpretation.

## 2. TS or ANCA Syndrome Reported Under the Diagnosis of “Myocarditis” Because of the Endo-Myocardial Biopsy (EMB) or CMR Imaging Findings

The histopathological changes of myocardial cell necrosis and mononuclear cell infiltration seen in certain time-related stages of TS or ANCA syndrome, and the CMR manifestations of “myocarditis-like features”, meet all the diagnostic criteria of myocarditis [23]. For this reason, TS or ANCA syndrome has been misinterpreted as “myocarditis” in many reports, as follows:

### 2.1 “Acute Focal or Multifocal Myocarditis” Reported During Catecholamine Administration and in Patients With Pheochromocytoma and Paraganglioma (PPGL)

Therapeutic or accidental catecholamine administration and diseases characterized by excessive catecholamine elevations, such as PPGL, are currently well-known trigger factors for TS [28, 29]. However, because of certain clinical presentations, ECG changes, CMR imaging features, and EMB findings that may be consistent with “myocarditis”, TS or ANCA syndrome triggered by catecholamine elevations has been reported as true “myocarditis” before and under the era of TS.

#### 2.1.1 Before the Takotsubo Era

For decades before the TS era, “catecholamine myocarditis” was reported as a complication of external catecholamine administration and of diseases associated with elevated catecholamine levels, such as PPGLs [30, 31]. The diagnosis of “PPGL-induced myocarditis” was based on the clinical picture and ECG changes [32], histopathological features through either EMB [18] or autopsy [30], and CMR imaging findings [20]. Van Vliet *et al*. [30] reported that 15 (58%) of 26 patients who died due to pheochromocytoma revealed disseminated focal myocardial lesions, which the investigators deemed as “active catecholamine myocarditis”. Jepson *et al*. [31] reported on two deaths associated with previously unsuspected pheochromocytoma. Post-mortem examination revealed “myocarditis”, which the authors assumed was the cause of cardiac arrhythmias leading to the death of the patients. The cardiac histopathological feature in patients with PPGLs is focal contraction band necrosis, accompanied by inflammatory cell infiltration and fibrosis [33].

#### 2.1.2 During the Takotsubo Era

Cases with findings consistent with PPGL-induced TS have been published under the “myocarditis” diagnosis [18, 19, 20]. Baratella *et al*. [18] reported a case of a 25-year-old woman with pheochromocytoma-induced reversible left ventricular dysfunction, in which the EMB revealed diffuse interstitial inflammatory cell infiltration, predominance of lymphocytes, and myocardial necrosis; the patient had normal coronary arteries. The case was reported under “an unusual case of myocarditis”. After careful review of the pattern of left ventricular dysfunction and its reversibility within weeks in that case, it is justified to deem that case as pheochromocytoma-triggered TS. Another case with pheochromocytoma and CMR imaging findings consistent with “acute myocarditis” at the basal segments of the left ventricle has been reported [20]. However, the ECG findings of widespread ST depressions and the echocardiographic and CMR imaging findings of basal segment hypokinesis argue strongly for a basal TS pattern (inverted TS) with LGE at the basal segments. Rostoff *et al*. [19] reported on a patient with PPGL presenting with left ventricular dysfunction and cardiogenic shock. The condition was deemed “fulminant adrenergic myocarditis” based on clinical, laboratory, and CMR imaging findings, which revealed myocardial oedema in the lateral, inferior, and posterior walls. In patients with PPGLs undergoing CMR imaging, cardiac involvement is frequently detected as changes consistent with “myocarditis” (focal and diffuse fibrosis) and left ventricular dysfunction [34]. Using CMR imaging, Ferreira *et al*. [34] demonstrated impaired left ventricular function (ejection fraction <56%) in 38% (11 out of 29) of patients with pheochromocytoma. The peak systolic circumferential strain and diastolic strain rate were also impaired. The patients also had higher myocardial T1, areas consistent with “myocarditis” and focal fibrosis on CMR imaging. Left ventricular dysfunction returned to baseline post-surgery; however, impairment of systolic and diastolic strain rate, and some fibrosis, persisted.

### 2.2 Interpretation of Some of the EMB or CMR-Imaging Findings in TS as True “Myocarditis” Have Excluded the Mother Diagnosis (TS) Because of the Diagnostic Criteria for TS

In many of the reported diagnostic criteria for TS, including the modified and revised ones, the diagnostic term of “myocarditis”, irrespective of the underlying cause of the “myocarditis features”, was regarded as an exclusion criterion for TS. Myocarditis was one of the exclusion criteria in the first proposed criteria of TS presented by Abe and Kondo [35] in 2003. One year later, Bybee* et al*. [36] reported on Mayo Clinic diagnostic criteria, in which myocarditis was one of the exclusion criteria for TS. Subsequently, the inclusion of myocarditis as an exclusion criterion for the diagnosis of TS has been retained and carried forward in a “hand-me-down” manner in new, modified, and revised criteria [37], except in the Johns Hopkins criteria [38], where myocarditis appropriately disappeared as an exclusion criterion. This criterion has resulted in the erroneous exclusion of TS in two ways: first, the “secondary myocarditis-like features” that TS may manifest have been misdiagnosed as myocarditis, thereby excluding TS diagnosis. Second, countless physical stress factors may trigger TS [39] and among others is that the severe viral, bacterial or other infectious diseases, which may cause myocarditis, are also strong physical stress factors that may trigger TS just as any other stress factors as sepsis and other critical illnesses [40]; TS may have been excluded because of the myocarditis criterion.

### 2.3 Typical Cases of TS Reported as “Myocarditis” Because of the EMB or CMR Imaging Findings

Cases with typical TS and with myocardial histopathological findings consistent with “acute myocarditis” have also been reported. Four years before the introduction of the term takotsubo, the Case 18–1986 [21], case records from the Massachusetts general hospital published in the New England Journal of Medicine, reported on a typical case of a broad left mid-ventricular ballooning pattern of TS in a 44-year-old woman who was admitted to the hospital because of crushing chest pain after she was informed that her 17-year-old son had committed suicide by hanging earlier in the day, *i*.*e*., typical broken heart syndrome. Coronary angiography revealed no coronary culprit lesion. The diagnosis of “myocarditis” was then confirmed by EMB, where myocardial cell necrosis and inflammatory cell infiltration were observed, and “catecholamine myocarditis” could not be excluded according to the authors. Caforio *et al*. [22] described a case of “acute biopsy-proven lymphocytic myocarditis mimicking mid-apical TS” in 2009. Histopathological analysis of EMB showed diffuse lymphomonocyte infiltration and myocyte necrosis, not typical for MI, but consistent with “Dallas diagnosis of active lymphocytic myocarditis”. CMR imaging showed signs of oedema and delayed gadolinium enhancement in the acute stage of the disease. Follow-up CMR imaging revealed normalization of the left ventricular function, disappearance of oedema, and no LGE. This case, as the authors have noted, is a typical case of TS; however, because of the biopsy findings, the case was deemed “acute biopsy-proven lymphocytic myocarditis mimicking takotsubo cardiomyopathy”.

Karamitsos *et al*. [41] described a case consistent with a mid-basal (inverted) left ventricular ballooning triggered by acute diabetic ketoacidosis precipitated by a persistent 7-day febrile illness related to viral upper respiratory infection in a 48-year-old man. CMR imaging confirmed mid-basal LVWMA, with signs of myocardial oedema in the same region. On the LGE imaging, there was patchy mid-wall hyperenhancement limited to the basal inferior and lateral wall, and the septum, typical of “viral myocarditis” according to the description by the authors. A follow-up CMR imaging after 1 month showed complete restoration of left ventricular function, significant regression of myocardial oedema, and persistence of areas of enhancement on LGE imaging. The authors deemed the case “acute myocarditis mimicking takotsubo cardiomyopathy”. However, after careful review of the clinical features, including the CMR imaging findings, one may conclude that the patient had typical inverted TS. The viral upper respiratory infection, together with the acute diabetic ketoacidosis, may have been the trigger factors.

Jorge *et al*. [42] reported a case of left ventricular mid-apical ballooning triggered by a stressful event in a 62-year-old woman. CMR imaging on the seventh day revealed that the LVWMA had already normalized. There was a hyperintense myocardial signal in T2-weighted turbo-spin echo sequences located in the lateral wall and LGE in the subepicardium of the same wall, findings also consistent with “myocarditis”. Consequently, all the above-described cases have typical TS, but because of EMB or CMR imaging findings, which are in fact manifestations of TS, these cases have been deemed “myocarditis”.

## 3. Evidence for the EMB and CMR-Imaging of “Myocarditis-Like Features” is Time-Related Evolutionary Secondary Changes and not Primary Myocarditis in TS or ANCA Syndrome

The most important reason for misdiagnosing TS or ANCA syndrome as “myocarditis” is the histopathological finding of myocardial cell necrosis and mononuclear inflammatory cell infiltration. These changes may be seen in TS or ANCA syndrome. To label this as myocarditis indicates that the myocarditis has caused myocardial cell necrosis. However, careful analysis of the sequence of events reveals that the process is the reverse: myocardial cell necrosis occurred due to another cause (incessant hypercontraction), which then elicited monocyte infiltration. Evidence supporting this approach and analysis follows:

### 3.1 The Histopathologic Process of TS or ANCA Syndrome at the Cardiac Sympathetic Nerve Terminals and the Evolutionary Time-Related Sequence of Changes

**Fig. 1.**
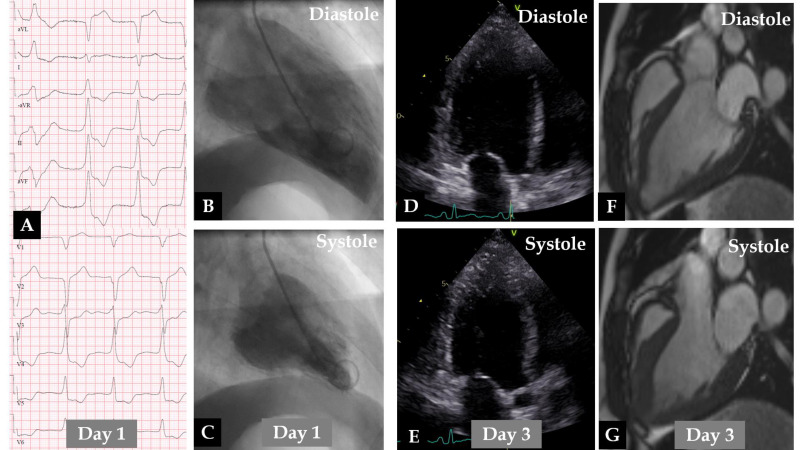
**Demonstration of a typical case of mid-basal (inverted) pattern of takotsubo syndrome (TS)**. The electrocardiogram (A) reveals sinus tachycardia with extensive ST depressions. Contrast left ventriculography (B,C) on day 1 shows akinesia/hypokinesia of the basal segments with good contractions of the apical half of the left ventricle. Echocardiography (D,E) and cardiac magnetic resonance (CMR) cine imaging (F,G) performed on day 3 show extensive hypokinesia of the mid-basal segments of the left ventricle, with good contractions only in the apical segments.

**Fig. 2.**
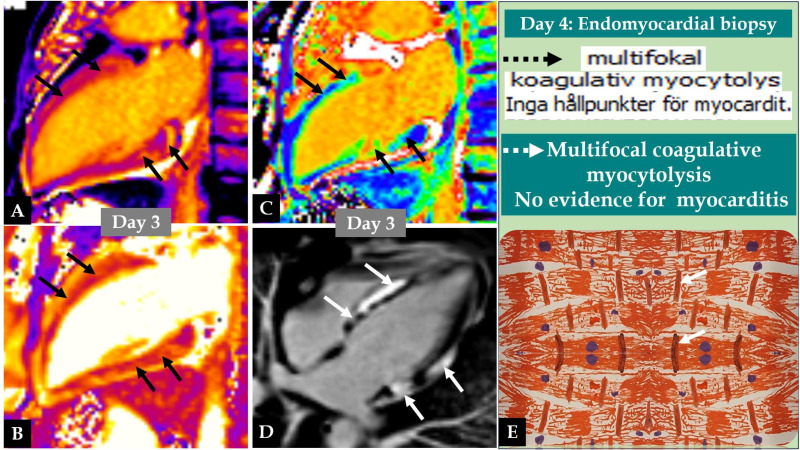
**Demonstration of the CMR images and the endomyocardial biopsy (EMB) findings of the same patient in Fig. 1**. CMR images on day 3 reveal clear signs of myocardial oedema with increased native T1 values in the mid-basal segments of the left ventricle (A, black arrows), increased T2 values in the corresponding segments of the left ventricle (B, black arrows), and signs of oedema in extracellular volume (ECV) images in the mid-basal segments of the left ventricle (C, black arrows). Late gadolinium enhancement (LGE) image of CMR reveals multifocal LGE in the mid-basal segments of the left ventricle (D, white arrows). One day after CMR imaging, EMB was performed and showed multifocal coagulative myocytolysis without evidence of acute myocarditis (E, written in Swedish, broken black arrow, and in English, broken white arrow). The cardiac coagulative myocytolysis lesion is drawn by hand, showing contraction band necrosis (E, white arrows) with intervening granulation and no evidence for inflammatory cell infiltration.

**Fig. 3.**
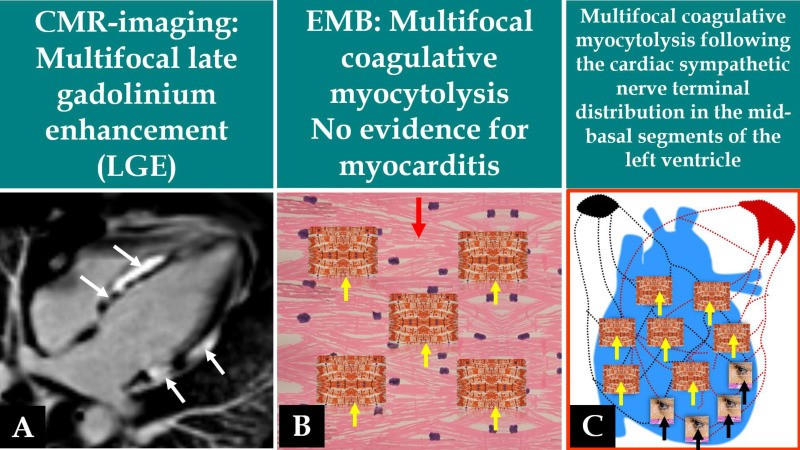
**Demonstration of the circumferential multifocal nature of both LGE during CMR imaging and the coagulative myocytolysis during EMB of the same patient in Figs. 1,2**. In this figure, the CMR image (A) and the EMB (B) show the multifocal nature of the disease process. The CMR image in this figure shows multifocal LGE in the mid-basal segments of the left ventricle (A, white arrows). The EMB shows multifocal coagulative myocytolysis (B, yellow arrows) amidst normal and viable myocardial cells (B, red arrow). The only anatomical cardiac structure that can explain the multifocal nature of coagulative myocytolysis lesions in this disease process is the cardiac sympathetic nerve terminals in the path of cardiac sympathetic nerves (C, yellow arrows). The cardiac sympathetic nerve terminals in the path of the cardiac sympathetic nerves in the apical region are normal (C, black arrows). Observe: (B,C) are drawn by hand.

TS or ANCA syndrome is a disease of the cardiac sympathetic nerve terminals [5, 43, 44, 45]. Substantial evidence indicates that in TS or ANCA syndrome, the sympathetic nervous system is hyperactivated, including cardiac sympathetic hyperactivation and excessive norepinephrine release and spillover at cardiac sympathetic nerve terminals [44, 46, 47]. The local release of large amounts of norepinephrine is known to stimulate the synthesis of adenosine 3-,5-cyclic phosphate, which, in turn, leads to the opening of calcium channels, resulting in a cellular influx of Ca^2+^ and an efflux of K^+^. This induces an actin–myosin interaction, which does not relax unless the calcium channels close [44, 46, 47]. The first histopathological change in TS or ANCA syndrome is the contraction bands, which represent a hypercontracted state of the myocardial cells with transverse bands of condensed myofilaments. Worth mentioning is that the “contraction bands” are a stage before “contraction band necrosis”, in which there are features of cell necrosis, including hypereosinophilia and loss of normal striations. The contraction bands may resolve without causing myocardial injury [6], and this process usually occurs in TS and explains the absence of LGE in most cases with TS. However, the contraction bands may be so severe in the acute stage of the disease that fragmentation and myocardial rupture may occur [48], or lead, in some cases, to so-called “stone heart” [49], where the heart will be irreversibly contracted. Another consequence is that the continuously high levels of norepinephrine may result in incessant hypercontracted sarcomeres and lead to myocardial cell death in a tonic state. This is the main underlying cause of the histopathological features of contraction band necrosis seen in some cases of TS or ANCA syndrome [44]. The incessant hypercontraction-caused myocardial cell necrosis elicits mononuclear cell infiltration and, thereafter, the healing process. Consequently, recognizing that mononuclear cell infiltration is a response or secondary process is fundamental and is not the primary cause of myocardial cell necrosis. This is one of the main reasons that TS or ANCA syndrome is misdiagnosed as “myocarditis”. The above-mentioned cardiac histopathological changes at the stage of mononuclear cell infiltration are not primary myocarditis; another term should have been used. This paper labels these changes as “secondary myocarditis-like features”, which is one of the manifestations of TS or ANCA syndrome. The second main reason for the misdiagnosis of TS or ANCA syndrome as “myocarditis” is the CMR imaging findings of hyperemia shown by early gadolinium enhancement/T1 weighted images, myocardial oedema shown by increased relaxation time/intense T-weighted images, and focal or multifocal non-ischemic necrosis/fibrosis shown by LGE [23]. These changes may be seen even when there are no histopathological alterations [50], or the histopathology exhibits only contraction band necrosis (coagulative myocytolysis) with no mononuclear cell infiltration as demonstrated in the three figures representing a patient with typical mid-basal TS pattern (inverted TS) documented by contrast left ventriculography, echocardiography, and cine CMR-imaging views (Fig. 1). The CMR imaging also revealed myocardial oedema and multifocal LGE at the mid-basal segments (Fig. 2). Since these CMR imaging findings exhibit oedema and multifocal LGE, the case was deemed as “myocarditis” despite absence of evidence for myocarditis by EMB performed one day after CMR imaging. The EMB revealed signs of multifocal coagulative myocytolysis without inflammatory cell infiltration. The circumferential pattern of both multifocal LGE and coagulative myocytolysis at the mid-basal segments is illustrated in Fig. 3.

### 3.2 Time-Related Evolution Phases of the Histopathological Features of TS or ANCA Syndrome Documented in Several Clinical Conditions Known to Trigger TS

The sequence of histopathological evolution in TS or ANCA syndrome is well documented in most known diseases that trigger TS. Baroldi and co-workers [51] reported on patients who died of MI and the changes that occurred in the myocardium in addition to the MI region. The authors state that, as an early change, hypercontraction/cross-bridging ensues. Subsequently, progressive destruction of myofibrillar remnants occurs, followed by monocyte/macrophage infiltration, leading to an alveolar pattern formed by empty sarcolemma tubes infiltrated by macrophages loaded with lipofuscin. This is followed by a healing phase with progressive collagenization ending in a fibrous scar. Greenhoot and Reichenbach [52] also studied and demonstrated cardiac autopsy of patients dying of SAH at different times after the onset of SAH. A patient died 18 hours after SAH. The cardiac histopathology showed focal disruption of myocardial cell cytoplasm with the formation of transverse contraction bands and intervening granularity, and no inflammatory reaction. In a patient dying 2.5 days following SAH, the autopsy showed foci of necrotic myocardial cells with myofibrillar degeneration and infiltration of the necrotic debris by mononuclear cells. This histopathological change has been interpreted as “myocarditis”. A patient died 14 days after the onset of SAH. Microscopically, the heart demonstrated multiple foci of myocytolysis with collapse of the supporting stroma and loss of myocardial cell cytoplasm without an inflammatory reaction or fibrous tissue proliferation. The same investigators [52] demonstrated that stimulation of the midbrain reticular formation in cats has produced lesions identical to those seen in patients with SAH and those created in rats by systemic administration of catecholamines. These investigators also stated that the lesions appeared most severe adjacent to intramyocardial nerves. The same histopathological pattern and time-related sequence of lesions in the form of contraction band necrosis, myocyte necrosis, followed by mononuclear cell infiltration occur in the dog myocardium following electric shock to the surface of the myocardium [53], in patients with pheochromocytoma and multiple endocrine neoplasia [54], and in patients with septic shock [55]. Consequently, it is of paramount importance to learn and recognize the sequence of histopathological changes in patients with TS or ANCA syndrome to avoid misinterpretation of secondary myocarditis-like features as true myocarditis.

### 3.3 CMR-Imaging Features Which Meet the Existent Diagnostic Criteria of “Myocarditis” Have Been Documented in Patients With Typical TS

Findings consistent with “myocarditis” or secondary “myocarditis-like features” have been reported both in studies and case reports in patients with TS. The CMR imaging revealed findings suggestive of “myocarditis” in 8 (13.6%) out of 59 patients with left ventricular apical ballooning and normal coronary arteries in a study reported by Eitel *et al*. [26]. In another study [50], during the acute phase of the disease, 5 of 15 patients with typical TS had LGE on CMR imaging. These changes were resolved completely on follow-up CMR imaging. Neil *et al*. [27] reported on the extent of myocardial oedema detected by CMR imaging at the acute stage and after 3 months in 32 patients with TS. The authors found that TS was associated with slowly resolving global myocardial oedema, the acute extent of which correlated with the regional LVWMA and with acute release of both plasma normetanephrine and NT-proBNP, but not with systemic inflammatory markers. The authors of these studies have concluded that in TS, there is an intramyocardial oedema secondary to a left ventricular inflammatory response to local cardiac sympathetic disruption and norepinephrine spillover. Gaikwad *et al*. [56] studied 44 TS patients using CMR imaging at a mean of 57 hours after admission and found that 18 patients (41%) had LGE localized to regions of LVWMAs. The signal intensity of LGE in TS patients was significantly lower than that of ST-elevation MI. The presence of LGE was associated with greater myocardial injury in the acute setting, but did not affect functional recovery. The pattern of LGE differed in TS patients from that seen in both ischemia and true myocarditis. LGE in TS patients involved the full thickness of the affected myocardium diffusely, rather than the subendocardial high signal observed with ischemic injury or the subepicardial layers typically seen in myocarditis. Follow-up CMR imaging at a mean duration of 8 months revealed full normalization of signal intensity and no residual LGE detectable by quantitative signal analysis.

Furthermore, several cases of the coexistence of TS and “myocarditis-like features” have been reported during the last 10 years; we have reported on the case of a 71-year-old woman with typical mid-apical TS triggered by both a physical and an emotional stress factor [57]. She also had CMR imaging findings of “myocarditis-like features”. Meanwhile, diffuse intramyocardial oedema was shown in the regions of left ventricular hypokinesia. Corresponding to the oedematous areas, there was increased contrast uptake both intramurally and in the subepicardial region, features consistent with “myocarditis”. There were no such changes in the basal regions of the left ventricle. These findings of “myocarditis-like changes” were essential features of TS; this has also been discussed elsewhere [58].

### 3.4 TS or ANCA Syndrome With CMR Imaging Finding of “Myocarditis-Like Features” but Negative Biopsy Findings for Myocarditis

Rolf *et al*. [50] studied 15 patients with TS with CMR imaging within 24 hours of admission. CMR imaging revealed LGE in five patients, which resolved completely during follow-up CMR imaging at 14 days. The signal intensity in the LGE sequences of the five patients was much lower than that seen in MI or myocarditis. The LGE was best seen in four-chamber views and short-axis slices. LGE appeared as multiple diffuse patches with intramural extension over the apical portion of the septal and lateral wall in a cougar-like pattern. Myocarditis was excluded by means of EMB, immunohistochemistry, and viral genome chain reaction. The authors concluded that LGE in TS is due to transient fibrosis and is caused by either necrosis or oedema. The three figures in this presentation demonstrate a typical case where Figs. 1,2 show a typical mid-basal pattern of TS with “myocarditis-like features” on CMR imaging; however, the EMB revealed multifocal coagulative myocytolysis with no evidence of myocarditis as demonstrated in Figs. 2,3.

## 4. The Louise Lake Diagnostic Criteria of “Myocarditis” Need Urgent Revision

Substantial evidence has been provided that TS or ANCA syndrome may, in certain stages of the disease process, have manifestations identical to those of “myocarditis” and may meet all the Louise Lake diagnostic criteria of myocarditis. However, scrutiny of the cardiac histopathological time-related evolutionary changes of the disease reveals that mononuclear cell infiltration always occurs after myocardial cell necrosis, which is caused by norepinephrine-induced incessant hypercontraction. Consequently, the “myocarditis-like changes” seen in TS or ANCA syndrome are secondary manifestations and not true myocarditis. This implies that the Louise Lake diagnostic criteria for myocarditis should consider these proofs and be revised to avoid misdiagnosis of TS or ANCA syndrome as true “myocarditis”.

## 5. Conclusion

TS or ANCA syndrome is a disease of the cardiac sympathetic nerve terminals. In some stages of the disease process, the condition has EMB and CMR imaging features consistent with “myocarditis”. For this reason, TS or ANCA syndrome has been and remains, at times, erroneously reported under the diagnosis of “myocarditis”. However, a critical analysis of the time-related evolutional histopathological changes reveals that the incessant hypercontracted sarcomere in TS or ANCA syndrome is the cause of myocardial cell necrosis, and the mononuclear cell infiltration occurs thereafter. Consequently, the EMB and CMR imaging features suggestive of myocarditis are secondary changes in TS or ANCA syndrome, not true myocarditis. This also implies that the Lake Louise diagnostic criteria for myocarditis should consider the sequence of events in TS or ANCA syndrome and be revised urgently.

## Abbreviations

ANCA, autonomic neurocardiogenic; CMR, cardiac magnetic resonance; ECG, electrocardiogram; EMB, endomyocardial biopsy; ECV, extracellular volume; LGE, late gadolinium enhancement; LVWMA, left ventricular wall motion abnormality; MI, myocardial infarction; PPGL, pheochromocytoma and paraganglioma; SAH, subarachnoid hemorrhage; TS, takotsubo syndrome.
